# Impact of sexual and reproductive health interventions among young people in sub-Saharan Africa: a scoping review

**DOI:** 10.3389/fgwh.2024.1344135

**Published:** 2024-04-18

**Authors:** Isaac Chipako, Saurabh Singhal, Bruce Hollingsworth

**Affiliations:** ^1^ Health Economics and Policy Department, Division of Health Research Graduate College, Lancaster University, Lancaster, United Kingdom; ^2^ Economics Department, Lancaster University, Lancaster, United Kingdom

**Keywords:** sexual and reproductive health, young people, adolescents, contraception, family planning, sub-Saharan Africa

## Abstract

**Objectives:**

The aim of this scoping review was to identify and provide an overview of the impact of sexual and reproductive health (SRH) interventions on reproductive health outcomes among young people in sub-Saharan Africa.

**Methods:**

Searches were carried out in five data bases. The databases were searched using variations and combinations of the following keywords: contraception, family planning, birth control, young people and adolescents. The Cochrane risk-of-bias 2 and Risk of Bias in Non-Randomized Studies-of-Interventions tools were used to assess risk of bias for articles included.

**Results:**

Community-based programs, mHealth, SRH education, counselling, community health workers, youth friendly health services, economic support and mass media interventions generally had a positive effect on childbirth spacing, modern contraceptive knowledge, modern contraceptive use/uptake, adolescent sexual abstinence, pregnancy and myths and misperceptions about modern contraception.

**Conclusion:**

Sexual and reproductive health interventions have a positive impact on sexual and reproductive health outcomes. With the increasing popularity of mHealth coupled with the effectiveness of youth friendly health services, future youth SRH interventions could integrate both strategies to improve SRH services access and utilization.

## 1 Introduction

Sexual and reproductive health (SRH) challenges are currently recognised through Sustainable Development Goal (SDG) number 3 (1, 2). SRH problems have been shown to account for about one-fifth of the disease burden worldwide (3) and the burden is much higher among young women in the reproductive age group (4). Sexual activity and experimentation are normative parts of adolescent development that may, at the same time, be associated with adverse SRH outcomes, including the acquisition of sexually transmitted illnesses (STIs), unplanned pregnancies and abortions (5, 6). Moreover, many young women are at high risk of lack of access to, and inconsistent or incorrect use of contraception leading to unintended pregnancies (7, 8). Unintended pregnancies result in disruptions in young women's education, professional opportunities and, essentially, reproductive sovereignty. Inequalities such as these have implications on a central pillar of the SDGs: to leave no one behind. While overall, significant progress has been made on the SDGs in years gone by, some discrepancies have continued, including those between rural and urban communities as well as those caused by socioeconomic status, gender, age and other demographic factors (9).

Young people especially in sub-Saharan African have been reported to have limited access to SRH services (10, 11). Access to SRH services is affected by a myriad of factors related to young people's SRH knowledge and awareness of availability of services, and access and usage of these services. Several cultural, socioeconomic and political factors further act as barriers to the delivery of SRH information and services to young people. Additionally, failure to provide youth friendly SRH services, unwelcoming behaviour and negative attitude by healthcare workers often act as barriers to young people's access and usage of SRH services (12, 13). These scenarios puts pressure on sub-Saharan Africa policy makers and practitioners to find ways of mitigating SRH challenges (14). Therefore, health care providers have an important role to play in ensuring that young people have access to high quality and non-judgmental SRH services in youth-friendly settings that recognize the unique bio-psychosocial needs of young people (6).

Several countries in sub-Saharan Africa including Zimbabwe, Malawi, Kenya, Rwanda, Ethiopia and South Africa have implemented successful SRH programs targeting young people (15, 16). Thus, African countries have acknowledged the importance of SRH among young people, and as a result, have been implementing related strategies both at community and facility levels. These strategies have included comprehensive sexuality education (CSE), referred to as sexuality and relationship education curricula that are age-appropriate and culturally relevant (17, 18). They have also encompassed peer education, mass media campaigns, cash transfers and youth-friendly centres—which are spaces created for young people to access SRH health information and services (19–21), and youth-friendly services—which are accessible and appropriate services that appeal to youths in a manner that promotes equity and interactions between users and providers (22).

## 2 Objective

The aim of this scoping review was to identify SRH interventions and provide an overview of the impact these interventions on reproductive health outcomes among young people in sub-Saharan Africa.

## 3 Methods

Preferred Reporting Items for Systematic reviews and Meta-Analysis extension for Scoping reviews (PRISMA-ScR) guidelines were used to search and select the articles included in this scoping review (23). To make sure that all relevant information was included in the analysis, the PRISMA-ScR checklist (Table 1) was utilised. Data extraction was also guided by the PROGRESS-Plus framework, which was suggested by the Campbell and Cochrane Equity Methods Group (24). The population, intervention, comparison, outcome and context (PICOC) model for review questions was applied in the designing of the research question (25).

**Table 1:** Search strategy in pubMed.

Search number	Search details	Results
25	((“Contraception”[Title/Abstract] OR “Contraceptives”[Title/Abstract] OR “family planning”[Title/Abstract] OR “family plan”[Title/Abstract] OR “birth control”[Title/Abstract] OR “birth prevention”[Title/Abstract] OR “planned parenthood”[Title/Abstract]) AND (“young person”[Title/Abstract] OR “young people”[Title/Abstract] OR “young adults”[Title/Abstract] OR “young adulthood”[Title/Abstract] OR “young women”[Title/Abstract] OR “young men”[Title/Abstract] OR “emerging adults”[Title/Abstract] OR “college students”[Title/Abstract] OR “Adolescents”[Title/Abstract] OR “Teenagers”[Title/Abstract] OR “Teenage”[Title/Abstract] OR “Teens”[Title/Abstract] OR “Youth”[Title/Abstract] OR “generation z”[Title/Abstract])) AND (2010:2023[pdat])	4,274
24	(“Contraception”[Title/Abstract] OR “Contraceptives”[Title/Abstract] OR “family planning”[Title/Abstract] OR “family plan”[Title/Abstract] OR “birth control”[Title/Abstract] OR “birth prevention”[Title/Abstract] OR “planned parenthood”[Title/Abstract]) AND (“young person”[Title/Abstract] OR “young people”[Title/Abstract] OR “young adults”[Title/Abstract] OR “young adulthood”[Title/Abstract] OR “young women”[Title/Abstract] OR “young men”[Title/Abstract] OR “emerging adults”[Title/Abstract] OR “college students”[Title/Abstract] OR “Adolescents”[Title/Abstract] OR “Teenagers”[Title/Abstract] OR “Teenage”[Title/Abstract] OR “Teens”[Title/Abstract] OR “Youth”[Title/Abstract] OR “generation z”[Title/Abstract])	10,851
23	“young person”[Title/Abstract] OR “young people”[Title/Abstract] OR “young adults”[Title/Abstract] OR “young adulthood”[Title/Abstract] OR “young women”[Title/Abstract] OR “young men”[Title/Abstract] OR “emerging adults”[Title/Abstract] OR “college students”[Title/Abstract] OR “Adolescents”[Title/Abstract] OR “Teenagers”[Title/Abstract] OR “Teenage”[Title/Abstract] OR “Teens”[Title/Abstract] OR “Youth”[Title/Abstract] OR “generation z”[Title/Abstract]	480,416
22	“generation z”[Title/Abstract]	237
21	“Youth”[Title/Abstract]	95,550
20	“Teens”[Title/Abstract]	7,545
19	“Teenage”[Title/Abstract]	10,043
18	“Teenagers”[Title/Abstract]	14,468
17	“Adolescents”[Title/Abstract]	241,992
16	“college students”[Title/Abstract]	26,817
15	“emerging adults”[Title/Abstract]	2,754
14	“young men”[Title/Abstract]	16,668
13	“young women”[Title/Abstract]	27,500
12	“young adulthood”[Title/Abstract]	9,827
11	“young adults”[Title/Abstract]	87,376
10	“young people”[Title/Abstract]	38,199
9	“young person”[Title/Abstract]	1,751
8	“Contraception”[Title/Abstract] OR “Contraceptives”[Title/Abstract] OR “family planning”[Title/Abstract] OR “family plan”[Title/Abstract] OR “birth control”[Title/Abstract] OR “birth prevention”[Title/Abstract] OR “planned parenthood”[Title/Abstract]	86,045
7	“planned parenthood”[Title/Abstract]	1,413
6	“birth prevention”[Title/Abstract]	311
5	“birth control”[Title/Abstract]	5,700
4	“family plan”[Title/Abstract]	18
3	“family planning”[Title/Abstract]	44,156
2	“Contraceptives”[Title/Abstract]	30,807
1	“Contraception”[Title/Abstract]	43,904

### 3.1 Search strategy

Five databases were searched: PubMed, Scopus, Psychological Information Database (PsycINFO), Cumulative Index to Nursing and Allied Health Literature (CINAHL), and the Cochrane Central Register of Controlled Trials. Predefined keywords, such as “contraceptives” and “young adults,” together with their synonyms, were used to search the databases. It was decided to create each concept's variations based on similar reviews. The initial search was conducted in August 2021 and updated in August 2023. Table 1 illustrates the search strategy for PubMed including Boolean operators, which was adapted for the other databases. Relevant articles were also searched using the PubMed “similar articles” function. To maximise the findings’ applicability to current policy, a literature search was conducted from January 2010 to August 2023.

### 3.2 Data collection and analysis

#### 3.2.1 Selection of studies

Two reviewers independently examined the titles and abstracts, excluding articles that were irrelevant. The articles that were identified were transferred to Mendeley Desktop, where any duplicated articles were eliminated. Afterwards, the reviewers assessed the eligibility of the remaining articles.

#### 3.2.2 Data extraction

Two reviewers independently gathered information from each article that was included in the comprehensive review by utilizing a predetermined excel spreadsheet form for data extraction. The excel spreadsheet form was developed by the reviewers. In case of any discrepancies, a third reviewer was involved to achieve a resolution. The following information was extracted from each article:

(1) Bibliographic information
(2) Study aims or questions.
(3) Study characteristics (design, sample size, number of arms)
(4) Intervention and control (type and characteristics of interventions and controls)
(5) Study setting (country)
(6) PROGRESS-Plus factors
(7) Outcome measures (type of outcome, definition of outcome)

#### 3.2.3 Criteria for considering studies for this review

Studies that met the following criteria were included:

Population: Studies that focused on youths aged 15–24 years in Sub-Saharan Africa. However, for the purpose and context of the scoping review the word youths was used interchangeably with young people.

Intervention: This review focused on articles that reported on the effectiveness of SRH interventions on pregnancy and contraceptive use. The review also focused on papers that report effectiveness of SRH interventions on secondary outcomes such as increased knowledge of contraceptives, positive attitude/change towards contraceptives and dispelling myths and misconceptions.

Comparison: Studies with comparison groups that included older people (25 and above), no intervention, standard care group and another intervention.

Study designs: Randomised controlled trials (RCTs), interrupted time series, prospective or retrospective cohort studies and controlled before and after designs that meet the inclusion criteria were considered for the study.

Outcomes:

Primary outcomes included studies with at least one of the following metrics:

• Using contraception; using a new technique; continuing or improving the usage of an existing method.
• Becoming pregnant (at least six months after the intervention started).

Secondary outcomes include the following:

• Attitude about contraception or a particular type of contraception.
• Knowledge of the effectiveness of contraceptives or the usage of effective methods.
• Adolescent sexual abstinence.

Context: sub-Saharan Africa.

Studies were excluded if:

• Full text and abstract were both unavailable or only the abstract was available but did not convey the needed data.
• Conference abstract
• Narrative or systematic reviews
• Published before January 2010

### 3.3 Assessment of study quality

The Cochrane Risk-of-Bias tool for randomised trials (RoB 2), version 2, was employed by the reviewers for randomised controlled trials (RCTs). Five categories were used to evaluate bias, with each aspect receiving a judgement (high, low, unclear), namely selection, performance, attrition, reporting, and other (26). Reviewers employed the Risk of Bias in Non-Randomized Studies—of Interventions (ROBINS-I) tool to evaluate the risk of bias in non-randomized controlled trials. Studies were categorised as having a low, moderate, significant, or critical risk of bias (27).

### 3.4 Data synthesis

Due to the heterogeneity in the design of the studies that were included, along with the diversity in outcomes and interventions, it was deemed unsuitable to conduct a meta-analysis. To enable the exploration of descriptive themes derived from the research, a thematic synthesis approach was employed for data synthesis. A narrative summary was utilized to provide an interpretation of the results and elucidate their connection to the objectives and inquiries of the review (28).

### 3.5 Patient and public involvement statement

Since the data for the review article was extracted from published articles, without direct patient involvement, ethical approval was not required.

## 4 Results

### 4.1 Identification of potential studies

**Figure 1:**
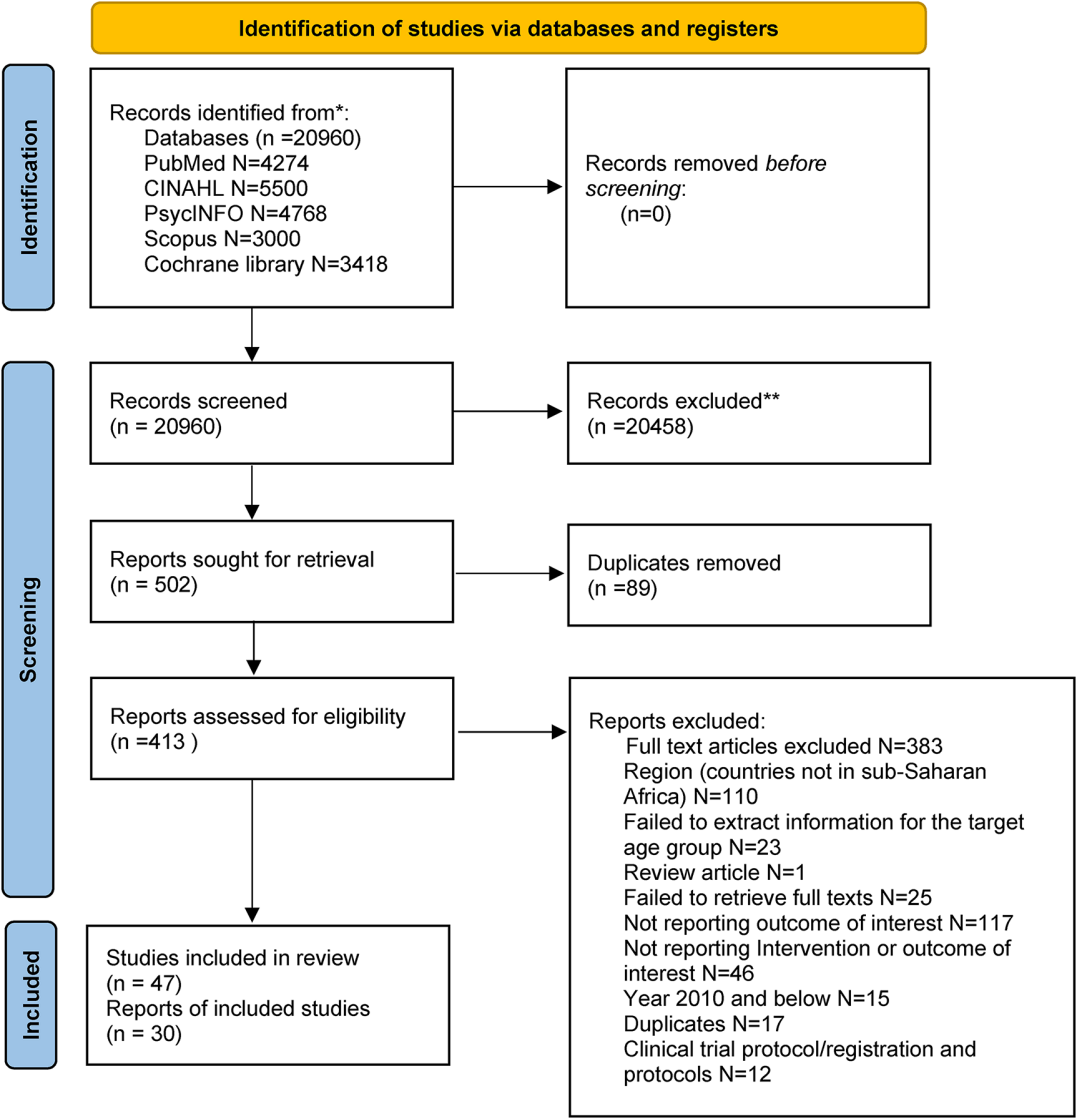
Flow chart of the studies identification and selection process.

Electronic searches of 5 databases identified 20,960 potential articles (Pubmed: 4,274, CINAHL: 5,500, Cochrane: 3,418, PsycINFO: 4,768, Scopus: 3,000). After 20,458 were excluded through screening the titles and abstracts, followed by removal of 89 duplicates, a total of 413 full text articles were screened for eligibility. Full text screening led to a total of 30 full text articles and 46 studies that were included in the scoping review. Figure 1 shows the flow chart of the studies identification and selection process.

### 4.2 Usage of PROGRESS-Plus factors

All 30 articles and 43 individual studies reported at least 2 PROGRESS-Plus factors (Tables 2, 3). Age distribution was the most reported PROGRESS-Plus factor (reported in 39 studies) followed by education levels, marital status, and parity which were reported in 35, 27 and 19 studies respectively. Religion was reported in 13 studies, and gender, occupation and socio-economic status were reported in 11 studies each. Place of residence and race/ethnicity were reported in 7 and 8 studies respectively. Living situation was the least reported PROGRESS-Plus factor, being reported in 3 studies. Most studies (*n* = 43) considered PROGRESS-Plus factors as control variables when measuring the effect of the intervention for example in logistic regression. Among these, age, education levels and marital status were the most controlled for. Three studies in an article by Morgan and colleagues (29) and one study in an article by Levy and colleagues (30) identified PROGRESS-Plus factors, but failed to include them in their final analyses.

**Table 2:** Characteristics of studies included in the review.

Author	Study design	Year	Location (Country)	Sample characteristics	Comparator	Outcome measures	Intervention details	Duration/length of intervention	Reported equity characteristic(s)
Morgan (29)	Non RCT (quantitative pre-test-post-test design)	2020	Nigeria	Non-pregnant first-time mothers (mean age 20.6), 63% were aged 20–24 years and 29% were aged 15–19 years. 224 participating partners	Pre-intervention	Increase birth spacing intentions	Community-based programs (Peer group sessions with first time mothers; small group sessions with the husbands/partners of peer group members; small group sessions with older women, typically the mothers or mothers-in-law of peer group members; home visits by Community health workers; community sensitization; and ongoing family planning service delivery at facilities and through mobile outreach).	4 months	None
Morgan (29)	Non RCT (quantitative pre-test-post-test design)	2020	Nigeria	Non-pregnant first-time mothers (mean age 20.6), 63% were aged 20–24 years and 29% were aged 15–19 years. 224 participating partners	Pre-intervention	Increase knowledge/awareness of modern contraceptive	Community-based programs (Peer group sessions with first time mothers; small group sessions with the husbands/partners of peer group members; small group sessions with older women, typically the mothers or mothers-in-law of peer group members; home visits by Community health workers; community sensitization; and ongoing family planning service delivery at facilities and through mobile outreach).	4 months	None
Morgan (29)	Non RCT (quantitative pre-test-post-test design)	2020	Nigeria	Non-pregnant first-time mothers (mean age 20.6), 63% were aged 20–24 years and 29% were aged 15–19 years. 224 participating partners	Pre-intervention	Decrease myths and misperceptions of using modern contraception	Community-based programs (Peer group sessions with first time mothers; small group sessions with the husbands/partners of peer group members; small group sessions with older women, typically the mothers or mothers-in-law of peer group members; home visits by Community health workers; community sensitization; and ongoing family planning service delivery at facilities and through mobile outreach).	4 months	None
Morgan (29)	Non RCT (quantitative pre-test-post-test design)	2020	Nigeria	Non-pregnant first-time mothers (mean age 20.6), 63% were aged 20–24 years and 29% were aged 15–19 years. 224 participating partners	Pre-intervention	Increase modern contraception use	Community-based programs (Peer group sessions with first time mothers; small group sessions with the husbands/partners of peer group members; small group sessions with older women, typically the mothers or mothers-in-law of peer group members; home visits by Community health workers; community sensitization; and ongoing family planning service delivery at facilities and through mobile outreach).	4 months	**Age (**15–19, 20–24, 25–29) **Gender** **Marital status** (Never married, living with partner/married, Divorced/separated/widowed) **Education levels** (Primary Junior Secondary, Secondary, Polytechnic, University) **Age of youngest child** **No. of living children** (0, 1, 2)
Brooks (45)	Non RCT (retrospective cross-sectional study)	2019	Niger	Young married women living in rural areas. Slightly over half (53.3%) of the women in the study population were older adolescents (18−19 years old) and about half (48.9%) had no formal education.	No intervention (No CHWs visits)	Increase modern contraception use	CHWs visits.	3 months	**Age** (13−15, 16−17, 18–19) **Education** (No school, Quranic school, Government school) **Parity/number of children** (0, 1, 2, 3**^+)^** **Race/ethnicity/tribe** (Zama, Hausa) **Occupation** **Place of residence**
Yakubu (31)	RCT	2019	Ghana	367 adolescent girls between the ages of 13–19 years. 185 in the intervention group and 182 in the control group.	Normal classes.	Improve adolescent sexual abstinence	Sexual Health Education (In addition to normal classes CSE was delivered to students for 1 month)	3 months	**Age** (years) (14–16, 17–19) **Social class** (Lower, Middle, Upper) **Ethnicity (**Dagombas, Gonjas**,** Ashantis**,** Others) **Religion (**Islam, Christianity)
Nuwamanya (35)	RCT	2020	Uganda	1,112 participants between the ages 18–30 years. The median age of participants was 21 years of age, and the majority were female (over 60%), unemployed (over 85%) and Christian (90%). Over 50% were resident in off-campus hostels and in a relationship.	Standard of care-SRH service	Increase contraceptives knowledge	mHealth (internet based mobile phone app for SRH service).	6 months	**Age** **Gender (**Male, Female) **Living situation** Campus hall, Off-campus hostel Rental home, Own home, Parent/Guardian home) **Residence (Hometown)** (Urban, Peri-urban Rural) **Marital status (**Relationship Single, Cohabiting, Married, Divorced, Widowed) **Employment (**Employed, Volunteer, Self-employed) **Religion (**Christian**,** Muslim, and Others)
Nuwamanya (35)	RCT	2020	Uganda	1,112 participants between the ages 18 and 30 years. The median age of participants was 21 years of age, and the majority were female (over 60%), unemployed (over 85%) and Christian (90%). Over 50% were resident in off-campus hostels and in a relationship.	Standard of care-SRH service	Increase use of modern Contraceptive	mHealth (internet based mobile phone app for SRH service).	6 months	**Age** **Gender (**Male, Female) **Living situation** Campus hall, Off-campus hostel Rental home, Own home, Parent/Guardian home) **Residence (Hometown)** (Urban, Peri-urban Rural) **Marital status (**Relationship Single, Cohabiting, Married, Divorced, Widowed) **Employment (**Employed, Volunteer, Self-employed) **Religion (**Christian**,** Muslim, and Others)
Ahmed (66)	Non RCT (Cross sectional study)	2020	Ethiopia	Women who were the age group between 15 and 24 years residing in rural areas (*n* = 4,061) and women who were the age group between 15 and 24 years residing in the urban area (*n* = 2,340)	No intervention	Increase use of modern Contraceptive	Mass Media Family Planning Messages (radio, television, newspaper/magazines, and mobile phones).	Cross sectional study	**Age** (15–19, 20–24) **Religion** (orthodox, catholic, protestant, Muslim, other) **Marital status (**single, married, separated, /divorced) **Education (**no education, primary, secondary, higher) **Wealth index** (poorest, poorer, middle, richer, richest,) **Region** **Parity/number of children** (0, 1–2, 3^+^)
Oberth (51)	Non RCT	2021	Zimbabwe	The mean age of participants was 15 years. The vast majority (91.17%) were adolescent girls (10–19 years old), with fewer (8.84%) young women (20–24 years old). Participants’ education ranged from none to tertiary level. Most (82.41%) were currently in school, while 17.60% were out of school or had never attended	Baseline vs. endline	Increase modern contraceptives knowledge	YFHS (Sista2Sista girls-only clubs create safe spaces for supporting and mentoring vulnerable AGYW) and Peer group SRH education.	12 months	**Age** (10–14, 15–19, 20–24 years). **Region/Province** (Harare, Manicaland, Mashonaland Central Mashonaland, East Mashonaland West, Masvingo, Matabeleland North, Matabeleland South, Midlands) **Education** (Never attended school, out of school, In primary school, In secondary school, In tertiary education) **Marital status** (Cohabitating Never married, Married Separated, Divorced, Widowed)
Oberth (51)	Non RCT	2021	Zimbabwe	The mean age of participants was 15 years. The vast majority (91.17%) were adolescent girls (10–19 years old), with fewer (8.84%) young women (20–24 years old). Participants’ education ranged from none to tertiary level. Most (82.41%) were currently in school, while 17.60% were out of school or had never attended	Baseline vs. endline	Pregnancy	YFHS (Sista2Sista girls-only clubs create safe spaces for supporting and mentoring vulnerable AGYW) and Peer group SRH education.	12 months	**Age** (10–14, 15–19, 20–24 years). **Region/Province** (Harare, Manicaland, Mashonaland Central Mashonaland, East Mashonaland West, Masvingo, Matabeleland North, Matabeleland South, Midlands) **Education** (Never attended school, out of school, In primary school, In secondary school, In tertiary education) **Marital status** (Cohabitating Never married, Married Separated, Divorced, Widowed)
Fikree (38)	Non RCT (Quasi-experimental)	2017	Ethiopia	20 youth friendly health units	Non-intervention YFHS	Increase contraceptive (LARCs) use	Counselling, YFHS and access to contraceptives (Counselling and access to all contraceptive methods provided by trained LARC YFHS providers in the same YFHS).	8 months	**Age** (years) (15–19, 20–24) **Marital Status** (Married, Living together, Single) **Parity** (Nulliparous, One Two or more)
Fikree (42)	Non RCT (Quasi-experimental)	2018	Ethiopia	20 youth friendly health units where peer educators referred clients	Non-intervention (One-day family planning refresher training that included LARCs)	Decrease Myths and Misconceptions about LARCs	Sexual Health Education (Proved by trained peer educators at YFHS units).	6 months	**Age** (years) (10–14, 15–19, 20–24, 25^+^) **Marital Status** (Married, living together, Single, Divorced/separated/widowed) **Parity** (None, 1–3). **Education** (Primary, Secondary, Technical/Vocational Training, University, Out of school, Others)
Lemani (37)	RCT	2017	Malawi	808 women mostly between 20 and 25 years, median age (22 years) and interquartile range (5 years). Most women were from the rural areas.	Family planning untrained CHWs and routine counselling	Increase modern family planning uptake among young women	Couples counselling and CHWs (Family planning trained CHWs and Couples counselling)	6 months	**Age** (14–19, 20–25, 26–30 years). **Education** (Never attended school, out of school, In primary school, In secondary school, In tertiary education) **Marital status** (Not married, Married) **Residence** (Urban, Rural) **Parity** (None, 1–3 children).
Almeida (49)	Non RCT (Quasi-experimental	2018	Angola	589 individuals included (mean age of 16.8 ± 2.5 years), 56.7% were males	Baseline vs. endline	Increase modern contraceptive knowledge among students	Sexual health education (Lectures with time for questions and answers, group work sessions and individual work)	2 months	**Gender** (Male, female) **Age** **Marital status** (Not married, married or marital)
Rosenberg J (52)	Non RCT	2018	Malawi	Female, 15–24 years old	Standard of Care consisting of vertical HIV testing, family planning, and sexually transmitted infection management in adult-oriented spaces, by providers without extra training.	Increase family planning service uptake	YFHS. Consisting of vertical HIV testing, family planning, and sexually transmitted infection management in an integrated youth-dedicated spaces and staffed by youth-friendly peers and providers.	12 months	**Age (year**) (15–17, 18–20, 21–24) **Marital status** (Single, Married, Divorced/widowed) **Education level** (Primary incomplete, Primary complete) Ever pregnant (No, Yes)
Wolf (39)	Non RCT	2017	Uganda	129 adolescents (ages 15–19)	Pre-intervention	Increase contraceptive knowledge	Reproductive health education (education program was taught as an interactive discussion)	3 weeks	**Gender** (Male,) Female) **Age** [Mean age 16.7 years (SD = 1.3)] **Marital status** (Unmarried) **Religion** (Catholic, Protestant, Muslim, Born again, Jewish, Orthodox, No Religion) **Education levels** Grade Level (S1, S2-S3, S4 23, S5-S6)
Gaughran (47)	Non RCT	2014	Kenya	42 female teenagers average age 16.5 (+/–1.31) years	Pre-intervention	Increase family planning knowledge of female teenagers.	Reproductive health education (which included didactic sessions, educational games, and open discussions)	6 weeks	**Age** (13–15, 16–17,>18) **Education levels** (Form 1, Form 2, Form 3)
Gaughran (47)	Non RCT	2014	Kenya	42 female teenagers average age 16.5 (+/– 1.31) years	Pre-intervention	Pregnancy	Reproductive health education (which included didactic sessions, educational games, and open discussions)	6 weeks	**Age** (13–15, 16–17,>18) **Education levels** (Form 1, Form 2, Form 3)
Hanne Keyser Hegdahl (48)	RCT	2022	Zambia	Adolescent girls mean age at baseline was 14.1 years (SD 1.34)	standard school and health services	Increase use of modern Contraceptive	CSE and economic support	2 years	**Age** **Wealth index** **Marital status** **Parity** **Education (**Highest level school parent/guardian**)**
Hanne Keyser Hegdahl (48)	RCT	2022	Zambia	Adolescent girls mean age at baseline was 14.1 years (SD 1.34)	standard school and health services	Increase use of modern Contraceptive	Economic support	2 years	**Age** **Wealth index** **Marital status** **Parity** **Education (**Highest level school parent/guardian**)**
Hanne Keyser Hegdahl (48)	RCT	2022	Zambia	Adolescent girls mean age at baseline was 14.1 years (SD 1.34)	standard school and health services	Increase in modern Contraceptive knowledge	CSE and economic support	2 years	**Wealth index** **Marital status** **Parity** **Education (**Highest level school parent/guardian**)**
Hanne Keyser Hegdahl (48)	RCT	2022	Zambia	Adolescent girls mean age at baseline was 14.1 years (SD 1.34)	standard school and health services	Increase in modern Contraceptive knowledge	Economic support	2 years	**Wealth index** **Marital status** **Parity** **Education (**Highest level school parent/guardian**)**
Michael T. Mbizvo (54)	RCT	2023	Zambia	986 adolescent girls aged 12–24 years from Solwezi and Mufumbwe	Routine CSE	Pregnancy	CHWs and CSE (To complement CSE community health workers provided information on available SRH services in schools)	3 years	**Age** (12–14, 15–19, 20–24) **Education** (Primary school and secondary school) **Religion** (Christianity and other)
Michael T. Mbizvo (54)	RCT	2023	Zambia	986 adolescent girls aged 12–24 years from Solwezi and Mufumbwe	Routine CSE	Pregnancy	YFHS and CSE.	3 years	**Age** (12–14, 15–19, 20–24) **Education** (Primary school and secondary school) **Religion** (Christianity and other)
Collins Annor (53)	Non-RCT	2021	Ghana	392 adolescents	community without YFHS	Increasing adolescent knowledge of contraceptives	YFHS	-	**Gender (**female, male) **Age (10–13, 14–15, 16–19)** **Place of residence** (Rural, urban) **Religion** (Catholic, other Christian, Islam) **Marital status of parents** (Single or married) **Education** (Education levels of breadwinner no formal education, basic education secondary and above)
Marlene Makenzius (40)	Non-RCT	2023	Kenya	1,368 school children mean (SD) ages were 16.4 years in the intervention group and 16.9 years in the control group.	Standard CSE	Decrease Myths and Misconceptions about LARCs	School based 8-hour stigma-reduction sexuality education over four sessions and standard CSE	1 month, 1 year	**Age** **Gender**
Wondimagegene (32)	RCT	2022	Ethiopia	224 sexually active secondary school adolescent girls aged 15−19 years		Increase modern contraception use	School-based per-led education	6 months	**Age** **Place of residence** **Education levels** **Religion** **Marital status**
Quraish Sserwanja (69)	Non-RCT	2022	Sierra Leone	Young women aged 15–24 years	No exposure to mHeath	Increase modern contraceptive uptake	mHealth	-	**Age (**15–19 and 20–24 years) **Residence** **(**Urban and Rural**)** **Region** (Northern, Eastern, Southern, Western and Northwestern) **Religion** (Muslims and Christians and others) (Level of education No education, primary, secondary and tertiary) **Wealth index** **(**Richest, richer, middle, poorer and poorest) **Parity** (None, one and above 1) **Marital status** (Married and Not married)
Quraish Sserwanja (69)	Non-RCT	2022	Sierra Leone	Young women aged 15–24 years	No exposure to mass media	Increase modern contraceptive uptake	Mass media (family planning messages on radio)	-	**Age (**15–19 and 20–24 years) **Residence** **(**Urban and Rural**)** **Region** (Northern, Eastern, Southern, Western and Northwestern) **Religion** (Muslims and Christians and others) **Level of education** No education, primary, secondary and tertiary) **Wealth index** **(**Richest, richer, middle, poorer and poorest) **Parity** (None, one and above 1) **Marital status** (Married and Not married)
Peter Gichangi (33)	RCT		Kenya	740 youth aged 18–24 years	standard care	Busting contraception myths and misconceptions	mHealth	7 weeks	**Age** (18–19 years 20–24 years) **Gender** (Male Female) **Education level** **(**Never gone to school Primary school Secondary school Postsecondary education**)** **Place of residence** **Marital status** (Single Friends with benefits/dating/cohabiting/engaged, Married) **Parity (**None One child, 2 + children**)**
Selema Akuiyibo (41)	Non-RCT	2021	Nigeria	8,930 young people aged 15–24 years	Pre-intervention	knowledge of condom use	MTV Shuga Peer Education	5 days	**Age** (15–19 20–24 years) **Gender** (Male Female) **Marital status (**Single, Married, Previously married) **Place of residence** **Highest educational attainment** (None vocational education, quranic education, primary education, secondary education, tertiary education, no response) **Employment status** [Student (In School) employed, unemployed**]** **Living situation** (Who respondent lives with parent or relative or alone or friends or partner)
Jay G. Silverman (34)	RCT	2023	Niger	Married adolescent girls aged 13−19	No intervention	Increase modern contraceptive uptake	CHWs	2 years	**Wife parity** (None One child, 2 + children) **Husband education (**Any modern, quranic only, no schooling) **Wife education (**Any modern, quranic only, no schooling)
Jay G. Silverman (34)	RCT	2023	Niger	Married adolescent girls aged 13−19	No intervention	Increase modern contraceptive uptake	Gender-segregated group discussion sessions	2 years	**Wife parity** (None One child, 2 + children) **Husband education (**Any modern, quranic only, no schooling) **Wife education (**Any modern, quranic only, no schooling)
Jay G. Silverman (34)	RCT	2023	Niger	Married adolescent girls aged 13−19 years old	No intervention	Increase modern contraceptive uptake	CHWs and gender-segregated group discussion sessions	2 years	**Wife parity** (None One child, 2 + children) **Husband education (**Any modern, quranic only, no schooling) **Wife education (**Any modern, quranic only, no schooling)
Ritah Bakesiima (57)	RCT	2021	Uganda	588 refugee adolescent girls aged 15−19 years.	Routine counselling, the standard of care.	Increase modern contraceptive acceptance	Peer counselling	Same day	**Age** (15−17, 18−19) **Religion [**Catholic, Anglican, Adventist, Other (Pentecostal, EFC, AIC)] **Ethnicity [**Acholi, Nuer, Dinka, Lotuho, Other (Shilluk, Luo, Bari)] **Education** (None, Primary Secondary, Tertiary) **Occupation** (Unemployed Employed/Selfemployed Peasant farmer, Student) **Marital status** (Single, Cohabiting, Married, Separated/Divorced/Widowed) **Parity**
Ritah Bakesiima (57)	RCT	2021	Uganda	588 refugee adolescent girls aged 15−19 years.	Routine counselling, the standard of care.	Decrease Myths and Misconceptions about modern contraceptives	Peer counselling	Same day	**Age** (15−17, 18−19) **Religion [**Catholic, Anglican, Adventist, Other (Pentecostal, EFC, AIC)] **Ethnicity [**Acholi, Nuer, Dinka, Lotuho, Other (Shilluk, Luo, Bari)] **Education levels** (None, Primary Secondary, Tertiary) **Occupation** (Unemployed Employed/Selfemployed Peasant farmer, Student) **Marital status** (Single, Cohabiting, Married, Separated/Divorced/Widowed) **Parity**
Nivedita L. Bhushan (55)	Non-RCT	2021	Malawi	Adolescent girls and young women aged 15–24 years.	Standard of care	Increased non-barrier contraception and condom uptake	YFHS	1 year	**Age** (15–19, 20–24 years) **Education level** (Completed primary, Did not complete primary) **Living Children** (Yes, no) **Marital status** (Single, ever married)
Nivedita L. Bhushan (55)	Non-RCT	2021	Malawi	Adolescent girls and young women aged 15–24 years.	Standard of care	Increased non-barrier contraception and condom uptake	YFHS ± Small group counselling sessions	1 year	**Age** (15–19, 20–24 years) **Education level** (Completed primary, Did not complete primary) **Living Children** (Yes, no) **Marital status** (Single, ever married)
Nivedita L. Bhushan (55)	Non-RCT	2021	Malawi	Adolescent girls and young women aged 15–24 years.	Standard of care	Increased non-barrier contraceptionand condom uptake	YFHS ± Small group counselling sessions ± Cash Transfer	1 year	**Age** (15–19, 20–24 years) **Education level** (Completed primary, Did not complete primary) **Living Children** (Yes, no) **Marital status** (Single, ever married)
Quraish Sserwanja (67)	Non RCT	2022	Zambia	3,000 adolescents aged 15–19 years	No access to mass media	Reduce teenage pregnancy	Mass media	-	**Age (**15–17 and 18–19 years**)** **Residence** **(**Urban and Rural**)** **Region** **(**Provinces**)** **Religion** **(Muslims and Christians and others)** **Level of education** **(**No education, primary, secondary and tertiary**)** **Wealth index** **(**Richest, richer, middle, poorer and poorest**)** **Parity (None, one and above 1)** **Marital status (Married and Not married)** **Working status (**Work, not working)
Marcy Levy (30)	Non RCT	2021	Kenya	384 pregnant adolescent girls, adolescent mothers aged 10–19 years	Pre-intervention	Increase modern contraceptive uptake	CHWs visits. (Home visiting team)	12 months	**Age** **Gender** **Marital status** (not consider in analysis)
Natsayi Chimbindi (68)	Non RCT	2023	South Africa	2,184 adolescent girls and young women aged 12–24 years	Pre-intervention	Increase modern contraceptive uptake	Mass media (MTV Shuga:Down South)	Baseline interviews were conducted between May 2017 and February 2018 and follow-up interviews April 2018 and September 2019	**Age** (13–14, 15–17, 18–19, 20–22) **Currently in school (No, Yes)** **Socioeconomic status (**Low, Middle, High**)** **Place of residence** (Urban/Periurban, rural)
Anjali Sharma (61)	Non RCT	2022	Zambia	1,627 young people with a median age of 22 (IQR 21–23) years	Pre-intervention	Increase knowledge of condom use	mHealth [Be in the Know Zambia (BITKZ) web application]	5 weeks	**Gender** (Female, Male) **Age** (18, 19, 20, 21, 22,23,24) **Marital status** (Single, married, divorced/widowed, prefer not to answer) **Education levels** (Less than secondary, secondary, vocational/technical, college/university) **Employment status** (Unemployed, student/trainee, part-time, full-time, prefer not to answer**)** **Wealth index** (Poor, average wealth, very wealthy)
Paul Macharia (62)	RCT	2023	Kenya	Adolescents aged 15−19 years randomly assigned to the intervention (*n* = 146) and control (*n* = 154)	Accessed SRH information from regular sources	Increase SRH knowledge	mHealth [Unstructured Supplementary Service Data (USSD)-based app]	3 months	**Gender** (Female, Male) **Education levels** (Primary, secondary and above)
Erhardt-Ohren (36)	RCT	2022	Niger	404 married adolescent girls	No intervention	Increase modern contraceptive and LARC utilization	Counselling (Small group discussions)	1 year 6 months	**Age** (13–­14, 15–­17, 18–­19 years) **Education level** (None, koranic only, government school) **Parity** (0, 1, 2+) **Tribe** (Hausa, Zarma, Fula) **Worked in last 12 months** (Yes, NO)
Erhardt-Ohren (36)	RCT	2022	Niger	404 married adolescent girls	No intervention	Increase modern contraceptive and LARC utilization	CHWs visits	1 year 6 months	**Age** (13–­14, 15–­17, 18–­19 years) **Education level** (None, koranic only, government school) **Parity** (0, 1, 2+) **Tribe** (Hausa, Zarma, Fula) **Worked in last 12 months** (Yes, NO)
Erhardt-Ohren (36)	RCT	2022	Niger	404 married adolescent girls	No intervention	Increase modern contraceptive and LARC utilization	CHWs visits and counselling (Small group discussions)	1 year 6 months	**Age** (13–­14, 15–­17, 18–­19 years) **Education level** (None, koranic only, government school) **Parity** (0, 1, 2+) **Tribe** (Hausa, Zarma, Fula) **Worked in last 12 months** (Yes, NO)

CHWs, community health workers; CSE, comprehensive sexuality education; YFHS, youth friendly health services; RCT, randomized controlled trials; LARCs, long-acting reversible contraceptives.

Bold values indicates the progress plus factors.

**Table 3:** Usage of PROGRESS-plus factors within all studies.

PROGRESS-Plus factor	Use of PROGRESS-Plus factors	Control variables in measuring intervention effect
Place of residence	7	7
Race/ethnicity	8	8
Occupation/employment status	11	11
Gender/sex	11	10
Religion	13	13
Education levels	35	35
Socio-economic status (SES)	10	10
Income	0	0
Number of living children/parity	19	19
Age	39	38
Marital status	27	26
Living situation	3	3

### 4.3 Risk of bias in included RCT studies

**Figure 2:**
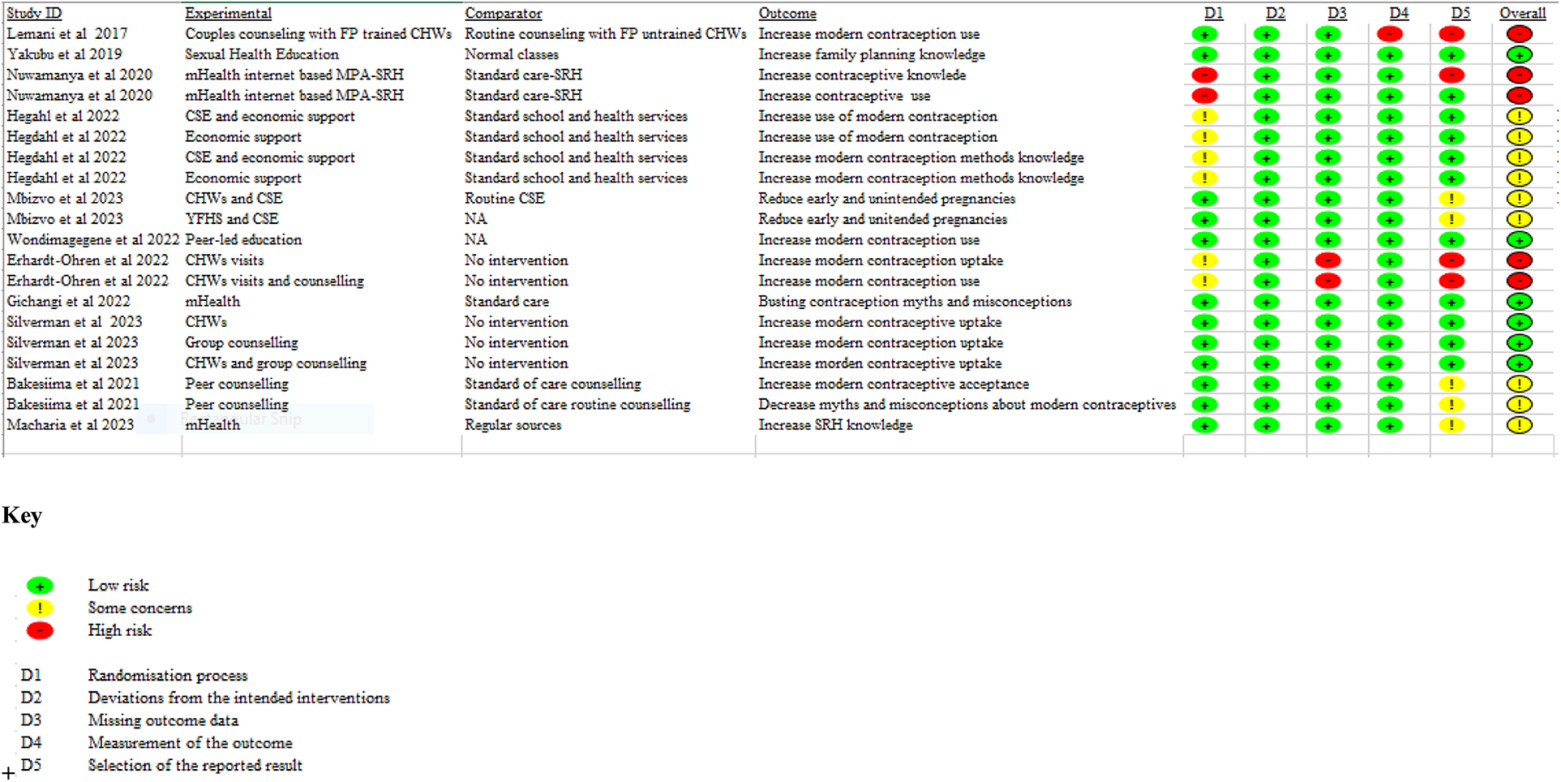
Risk of bias for each domain for RCT studies included in the review.

**Figure 3:**
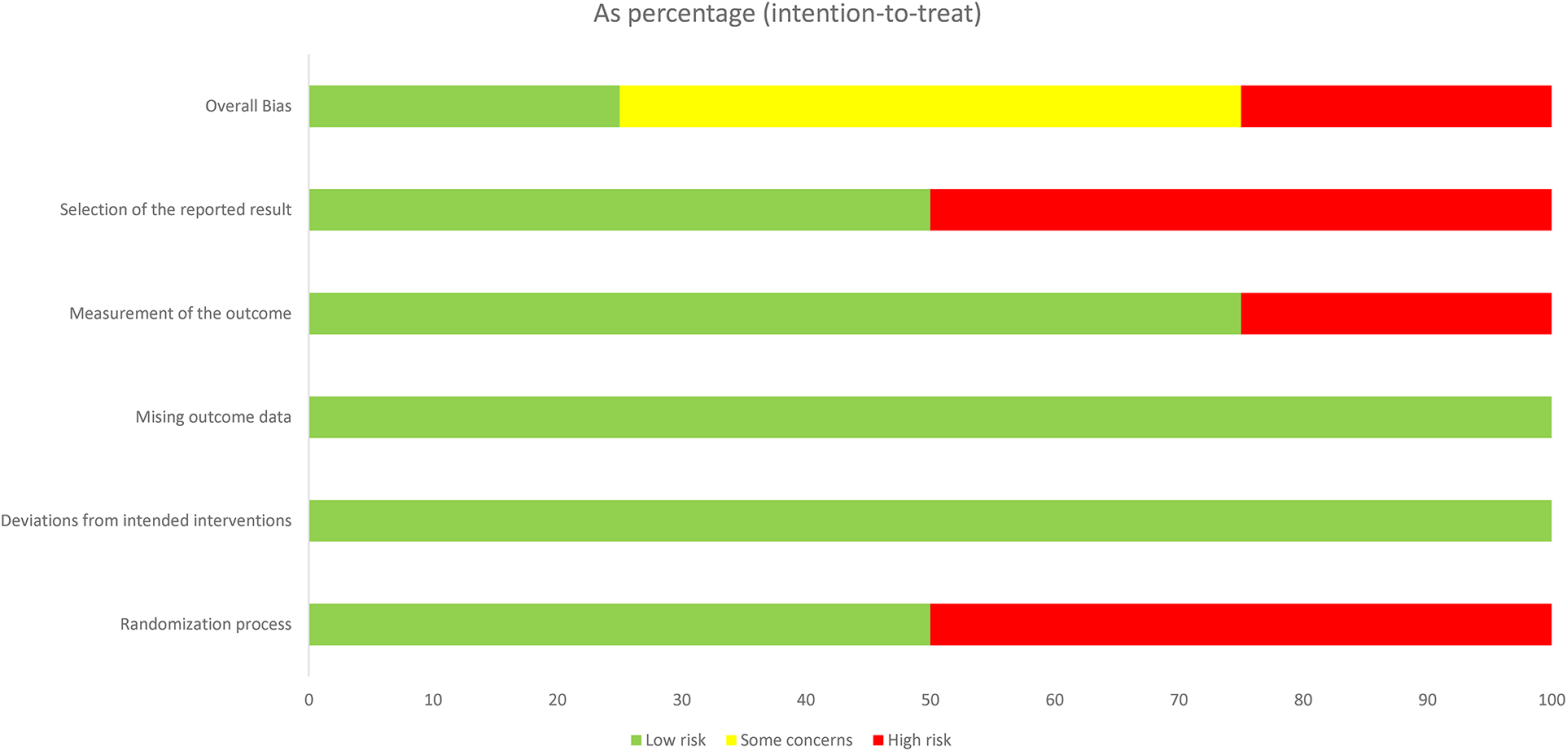
Risk of bias graph of each domain presented as percentages across all RCT included in the review.

The risk of bias results for RCT studies (*n* = 20) are summarised in Figures 2, 3. Reporting on the overall risk of bias domain, six studies had low risk bias, one study each from articles by Yakubu and colleagues, Wondimagege and colleagues and Gichangi and colleagues (31–33), and three studies from an article by Silverman and colleagues (34). Most of the studies 45% (*n* = 9) had some concerns in the overall risk of bias domain. Lastly, five studies, two studies each from articles by Nuwamanya and colleagues (35) and Erdhardt-Ohren colleagues (36) and one study from an article by Lemani and colleagues (37) had high risk of bias.

### 4.4 Risk of bias of non-randomised control studies included in the scoping review

**Figure 4:**
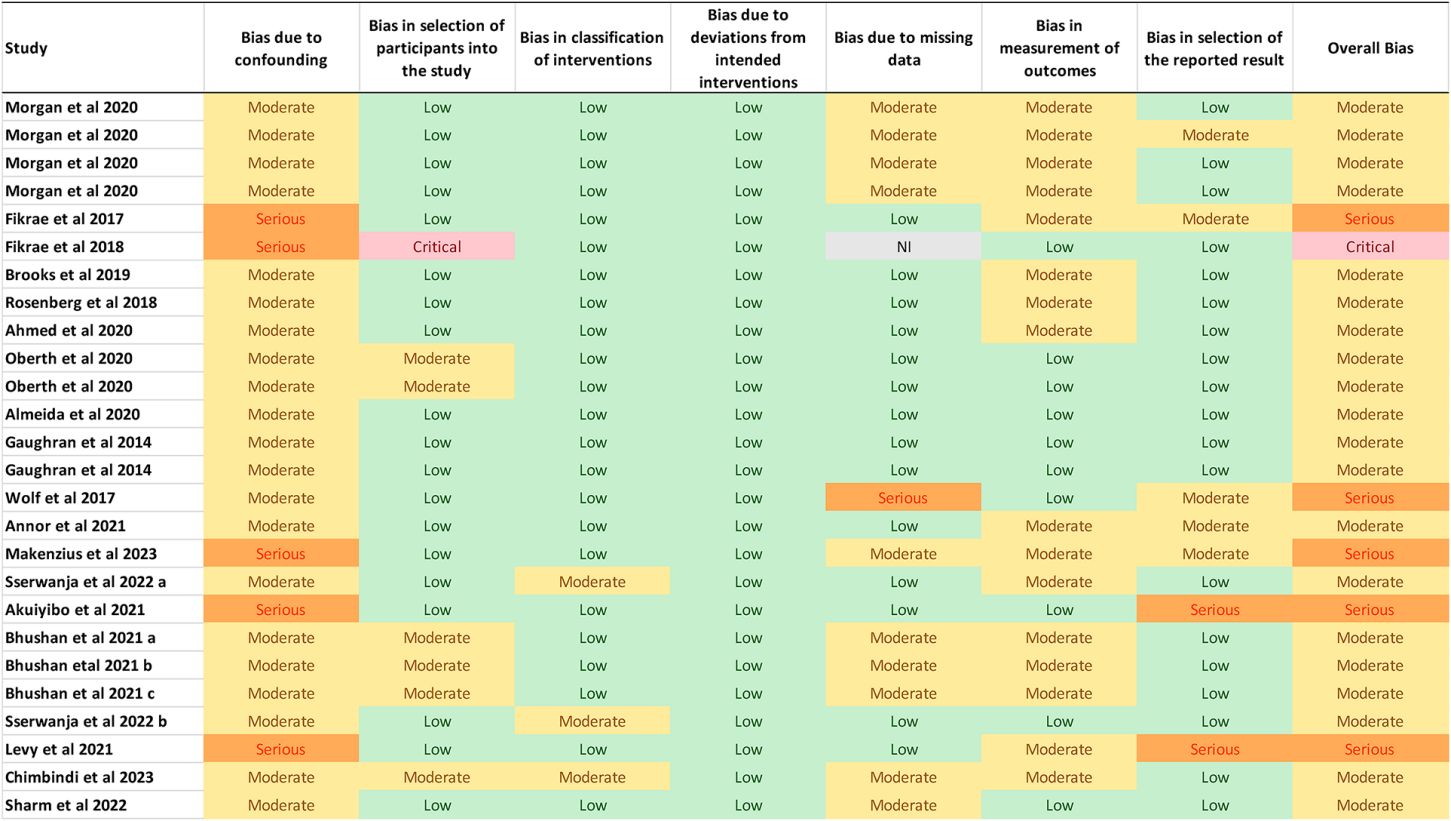
Quality assessment of quantitative non-randomised controlled studies included in the systematic review.

The risk of bias assessment results using the ROBINS-I tool for the non-RCTs studies (*n* = 26) is shown in Figure 4. Based on the ROBINS-I tool none of the studies included in the review had an overall low risk of bias. As expected with non-RCTs, most studies 76.9% (*n* = 20) included were labelled as moderate risk studies across all domains. Five studies were labelled as serious risk studies across all domains (30, 38–41) and one study by Fikree and colleagues (42) was judged to be critical risk of bias study.

### 4.5 SRH interventions identified from the review

The narrative synthesis of the results used in this scoping review was done in line with the recommendations set out in the PRISMA-ScR guidelines. Based on the research objectives, studies were classified into one of the following eight research domains: community-based program interventions, community health workers interventions, SRH education interventions, Youth friendly health services (YFHS) interventions, counselling interventions, mobile phone-based interventions, economic support, and mass media.

#### 4.5.1 Community-based program intervention

Four studies in an article by Morgan and colleagues 2020, used a community-based program with multiple interventions to improve SRH among young people. The program included home visits by community health workers, community sensitization, and continuing family planning service delivery at facilities and through mobile outreach. It also included peer group sessions with first-time mothers, small group sessions with the husbands or partners of peer group members, and small group sessions with older women, usually the mothers or mothers-in-law of peer group members (29).

#### 4.5.2 Community health workers interventions

Community health workers (CHWs) or lay health worker are defined as healthcare workers who perform functions related to health care delivery and are trained in some way in the context of an intervention, but who has not received a formal professional or para-professional certificate or tertiary education degree (7, 43). CHWs are an effective means to reach clients when access is limited especially in poor resourced remote rural areas (44). In the current review, 7 studies assessed the impact of CHWs interventions on SRH outcomes among adolescents and young adults. Brooks and colleagues, 2019 and Silverman and colleagues, 2023, reported that CHWs improved modern contraceptive uptake among adolescent girls (single, pregnant, married or pregnant) (34, 45). Mbizvo and colleagues, 2023, reported that CHWs coupled with comprehensive sexuality education (CSE) or YFHS improve pregnancy outcomes among adolescent girls. Lastly, Erhardt and colleagues, 2023, and Lemani and colleagues, 2017, reported that CHWs coupled with counselling improve modern family planning uptake among young women (36).

#### 4.5.3 SRH education

Comprehensive SRH education has been reported to be an effective strategy for improving young people's SRH outcomes (46). In the current review, 12 studies reported use of SRH education interventions as a strategy to improve SRH outcomes. Firstly, Yakubu and colleagues, 2019, and Guaghran and colleagues, 2014, reported that sexual health education improved sexual abstinence and pregnancy outcomes among adolescents girls (31, 47). Secondly, Fikree and colleagues, 2018, and Makenzius and colleagues, 2023, reported that SRH education provided by trained peer educators at YHFs and stigma-reduction sexuality education decreased myths and misconceptions about LARCs utilization (40, 42). One study from an article by Hegdahl and colleagues, 2022, reported that comprehensive sexuality education (CSE) combined with economic support improved modern contraceptives uptake among adolescent girls (48). Lastly, 5 studies reported that SRH education improved knowledge of modern contraceptive methods among young people (39, 41, 47–49). Details of the reproductive health education strategies utilized by each study are shown in Table 2.

#### 4.5.4 Youth friendly health services (YFHS) interventions

There is evidence that YFHS improve access to, and utilisation of SRH services by young people (19, 50). In the current review, 9 studies reported the use of YFHS interventions among young people. Four articles reported that YFHS interventions improved modern contraceptives knowledge and uptake by young people (38, 51–53). Oberth and colleagues, 2021, and Mbizvo and colleagues, 2023, reported that YFHS combined with peer group education and CSE, respectively, improved pregnancy outcomes among adolescent girls (51, 54). Three studies from an article by Bhushan and colleagues, 2021, reported that YFHS interventions improved non-barrier contraceptives and condoms uptake among young women (55) (see Table 2 for details of the interventions).

#### 4.5.5 Counselling interventions

Counselling, as an intervention, has been shown to improve SRH services utilisation among young people. Counselling can be delivered directly in person, online, or via the telephone, either by medical or nursing staff, or peers, in individual or group settings. The counselling interventions may consist of a single component or multiple components delivered in a single session, or in multiple sessions at various time points (56). In the present review, 10 studies utilized counselling interventions as strategies for improving modern family services uptake and decreasing myths and misconceptions about modern contraceptives. Among these studies, 2 from an article by Bakesiima and colleagues, 2021, reported that peer counselling improved modern contraceptives acceptance and dispelled myths and misconceptions about modern contraceptives among adolescent girls aged 15–19 years (57). Furthermore, 8 studies from 5 articles reported that groups or couples counselling only, and counselling combined with different SRH interventions (including CHWs, economic support and YFHS) increased modern family planning services uptake and utilization among young women (34, 36–38, 55) (see Table 2 for details of the counselling multicomponent interventions).

#### 4.5.6 Mobile phone-based interventions (mHealth)

Expansion of mobile phone technology and use in recent years provides an important tool to reach underserved populations in low to middle income countries. Populations with restricted access can be reached despite location and need (58–60). With the increasing popularity of mobile based interventions with young people, they promise to improve SRH services utilisation young people. In the present review, four studies utilised mHealth interventions to improve SRH outcomes (33, 35, 61, 62). Nuwamanya and colleagues, 2020, used an internet based mobile phone application to improve the use of modern contraceptive methods (35). Gichangi and colleague, 2022, reported that there was a statistically significant drop in the average absolute number of contraceptives myths and misconceptions believed by the mHealth intervention arm between baseline and endline (33). Sharma and colleagues 2022, reported that at the endline, an mHealth intervention resulted in higher level of knowledge related to condoms and on wearing condoms correctly (61). According to Macharia and colleagues, (2023), there was a statistically significant difference in the total knowledge scores in the mHealth intervention group compared with the control group. Young people reported gaining knowledge on abstinence and condom use from an mHealth application (62).

#### 4.5.7 Economic support

Gender inequality and economic constrains are central factors limiting young women's urgency regarding their own SRH, and ability to use their preferred contraceptive methods (48, 63). Economic support including cash transfers have been theorised to reduce young women's economic vulnerability and engagement in unsafe or asymmetric transactional sex (48, 64). Moreover, free access to a broad contraceptive method mix could increase contraceptive uptake, reduce unmet need, and increase agency in contraceptive decision-making among young women in resource-limited settings (63). In the present review, Hegdahl and colleagues, (2022), reported that there was no evidence of the effects of economic support on contraceptive use among those ever sexually active. However, the addition of CSE improved modern contraceptive use and knowledge of modern contraceptives compared to economic support alone among those recently sexually active (48). Likewise, Bhushan and colleagues, 2021, reported that cash transfers combined with YFHS and small group counselling increased non-barrier contraception and condom uptake (55). In conclusion, economic support coupled with other SRH interventions improves reproduction health outcomes among young women.

#### 4.5.8 Mass media

Mass media campaigns have the potential to effectively convey SRH messages to a wide population. Due to their ability to reach the masses, these campaigns can specifically target a significant number of and young individuals at a relatively minimal expense. Mass media campaigns typically utilize various platforms such as newspapers, television, radio, magazines, social media, and billboards within sub-Saharan Africa. Additionally, they can also be executed through cinema or emerging digital media channels, which encompass websites, pop-up and banner advertisements, codes, and viral marketing (65). Despite previous research highlighting that mass media campaigns can influence SRH (41, 66–68), there have been few attempts to synthesise evidence across young people's reproduction SRH outcomes. Similar to the review by Stead and colleagues, 2019, this study reported mixed evidence of the effect of mass media campaigns on SRH outcomes (65). After adjusting for covariates, Sserwanja and colleagues, 2022, reported that hearing family planning messages on radio and reading texts on mobile phones were statistically associated with increased modern contraceptives uptake among young people in Sierra Leone (69). They further reported that young women who had exposure to family planning messages on radio and mobile phones were more likely to use modern contraceptives when compared to their counterparts without the same mass media exposure. In a different study conducted in Zambia, Sserwanja (67), reported that adolescent girls who had daily access to magazines or newspapers, or internet were less likely to be pregnant or to have had a pregnancy compared with those without the same mass media exposure. After adjusting for HIV-prevention, intervention-exposure, age, education, socioeconomic status, Ahmed and colleagues, 2020, reported that MTVShuga-DS exposure was associated with increased modern contraceptives uptake and consistent condom use among young Ethiopians (66). However, Ahmed and colleagues, 2020, reported that there was no statistically significant association between young women exposed to mass media family planning messages and modern contraceptives uptake in rural areas.

## 5 Discussion

According to the review, a variety of comprehensive interventions aimed at promoting and providing consistent birth control methods, sexual health education, counselling, and other related services may be able to prevent and control the negative effects associated with risky sexual behaviour among young people in sub-Saharan Africa. It has been demonstrated that raising awareness of SRH and the use of contraceptives lowers the number of unintended births among people. Our findings align with previous assessments that assess the efficacy of different treatments in enhancing teenage self-reported health, and integrate several interventions under a more comprehensive framework to assess their combined effectiveness. A combination of educational and contraceptive interventions may help reduce the rate of unintended pregnancies among adolescents, according to a Cochrane review on primary prevention interventions (school-based, community or home-based, clinic-based, and faith-based). On the other hand, the data from that study showed conflicting results for secondary outcomes, such as the onset of sexual activity, the use of birth control, abortion, childbirth, and STIs (70). Group-based comprehensive risk reduction intervention was found to be an effective technique to lower adolescent pregnancy, HIV, and STIs (71).

Thus, it is essential to raise knowledge of the advantages of contraceptive services and empower young people to make their own decisions about taking contraceptives (72). Combining educational programs in communities and schools with YFHS, health centre outreach initiatives, and media campaigns are examples of interventions with supporting data (73). Reproductive health services are more likely to be accessed when initiatives to increase service quality are combined with community outreach to encourage young people SRH (19). Regarding services, a number of program evaluations have detailed challenges that many teenagers encounter, including judgmental provider attitudes, a lack of anonymity, a dearth of alternatives for contraception, and a lack of rules and procedures to safeguard teenagers’ rights to information and services (21). Approximately one out of every five nations in the world has official rules that restrict access to contraceptive services: Among the most prevalent limitations, parental consent requirements are in place in 9% of the 186 countries for which data is available; limits based on a minimum age or marital status are in place in 5% of nations (74). Nevertheless, teenagers continue to experience provider prejudice in a variety of ways even in nations without official limits. Because of erroneous concerns that hormone treatments might interfere with a young person's ability to conceive, physicians might not advise hormonal treatments to them, or they might discriminate against single youth because they think they shouldn't engage in sexual activity (75). Recent guidelines for self-care treatments, such over-the-counter oral contraceptive tablets and self-administered injectables, may be able to assist young people in overcoming some of these fundamental obstacles (76).

### 5.1 Limitations and recommendations

Despite providing a broad overview of the impact of SRH interventions on reproductive health outcomes among young people in sub-Saharan Africa, the focus on a scoping review limited our ability to examine the impacts of interventions in detail and statistically. Statistical synthesis was not possible due to considerable heterogeneity across the large numerous articles and studies included in the review, and the SRH outcomes and interventions reported. To perform a meta-analysis, we recommend that future reviews should focus on one SRH outcome and one intervention.

Practice of medicine and public health interventions supported by mobile devices are effective strategies for improving reproductive health outcomes among young people as they promote SRH services utilization (33, 35, 61, 62). This is partly due to their popularity among young people, privacy, and ability to reach populations with restricted access to direct SRH services (58–60). Mass media campaigns can be utilized to communicate SRH information in mass populations and results from this review highlights that they can improve reproductive health outcomes. Since they are delivered at population level, mass media campaigns can target numerous numbers of young people at relatively low costs (65, 77, 78). Harnessing the advantages of both mHealth and mass media intervention could result in development of low cost, easily accessible, convenient, and age-appropriate strategies for widespread dissemination of SRH information (79). Therefore, it is highly recommended to integrate both mHealth and mass media campaigns in future SRH interventions targeting young people with access to mobile devices.

Despite different PROGRESS-Plus factors being reported to influence the effect of SRH interventions, studies in the review did not include all PROGRESS-Plus factors in their analysis which might have resulted in over estimation or underestimation of the impact of the interventions. Therefore, we recommend future studies with rigorous designs and longer-term follow-up to use all PROGRESS-Plus factors as control variables to measure the impact of SRH interventions and maximize applicability of results.

### 5.2 Contribution of the findings to the field of study

Young people make up a big proportion of the population in Africa's developing economies, with approximately 20% of the population aged 15–24 years. Despite increased attention to family planning, young people in this region continue to face numerous SRH challenges. The review findings could guide future strategies to improve SRH services’ access and utilization among young people in sub-Saharan Africa thereby protecting them from unintended pregnancies and unsafe abortions. The review suggests that community-based programs, mHealth, SRH education, counselling, community health workers’ visits and youth friendly health service interventions generally had a positive effect on child spacing, modern contraceptive knowledge, modern contraceptive use, adolescent sexual abstinence, pregnancy and myths and misperceptions of modern contraception. Evidence from the review has shown that bringing awareness of the benefits of modern contraceptives and enabling young people to make their own decisions regarding contraceptive services is vital. Several studies reported that mHealth is effective in promoting SRH services utilization. Therefore, future SRH strategies could utilise mHealth to improve knowledge, access and uptake of SRH services. Due to their ability to ensure privacy and reach underserved populations, incorporating mobile devices into SRH interventions among young people and utilizing mass media campaigns to reach a wider audience are recommended strategies. Combining these two components in future national SRH interventions has the potential to improve outcomes, positively impacting reproductive health on a larger scale, at relatively lower costs.

## 6 Conclusion

Community-based programs, mHealth, SRH education, counselling, CHWs, YFHS, economic support and mass media interventions generally had a positive effect on childbirth spacing, modern contraceptive knowledge, modern contraceptive use, adolescent sexual abstinence, pregnancy and myths and misperceptions of modern contraception. This scoping review could inform administrators, managers, and policymakers on the different SRH interventions to implement in different settings.

## Data Availability

The original contributions presented in the study are included in the article/Supplementary Material, further inquiries can be directed to the corresponding author.
